# Albendazole metabolites excretion in human saliva as a biomarker to assess treatment compliance in mass drug administration (MDA) anthelmintic programs

**DOI:** 10.1038/s41598-024-56804-w

**Published:** 2024-03-15

**Authors:** E. Nieves, R. Cimino, A. Krolewiecki, M. Juarez, C. Lanusse, L. Alvarez, L. Ceballos

**Affiliations:** 1 Facultad Regional Orán, Instituto de Investigaciones de Enfermedades Tropicales, Universidad Nacional de Salta, Orán, Salta Argentina; 2 Laboratorio de Farmacología, Centro de Investigación Veterinaria de Tandil (CIVETAN), UNCPBA-CICPBA-CONICET, Tandil, Buenos Aires Argentina; 3 Facultad de Ciencias Veterinarias, Universidad Nacional del Centro de la Provincia de Buenos Aires (UNCPBA), Tandil, Buenos Aires Argentina

**Keywords:** Albendazole, Soil transmitted helminths, Saliva, Microbial communities, Parasitology

## Abstract

Soil-transmitted-helminth (STH) infections continue to be a persistent global public health problem. Control strategies for STH have been based on the use of mass drug administration (MDA). Coverage and compliance assessment is critical to understanding the true effectiveness of albendazole (ABZ) in those MDA programs. The aims of this work were to characterize the pattern of albendazole and metabolites excretion in human saliva, and to develop a saliva-based biomarker (HPLC drug/metabolite detection) useful to accurately estimate the coverage/compliance in MDA campaigns. The study subjects were 12 healthy volunteers treated with a single oral dose of ABZ (400 mg). Saliva and blood (dried blood spot, DBS) samples were taken previously and between 2 and 72 h post-treatment. The samples were analyzed by HPLC with UV detection, C_18_ reversed-phase column. ABZ sulphoxide was the main analyte recovered up to 72 h p.t. in blood and saliva. The concentration profiles measured in the blood (DBS samples) were higher (*P* < 0.05) than those in saliva, however, this ABZ-metabolite was recovered longer in saliva. The in vivo measurement of drugs/metabolites in saliva samples from ABZ-treated volunteers offers strong scientific evidence to support the use of saliva as a valid biological sample for assessing compliance in MDA programs.

## Introduction

Neglected tropical diseases (NTDs) refer to a group of illnesses that mainly affect individuals living in poverty-stricken regions^1^. Soil-transmitted helminth (STH) infections caused by *Ascaris lumbricoides*, *Trichuris trichiura*, *Strongyloides stercoralis* and hookworms (*Ancylostoma duodenale* and *Necator americanus*), are among the most common NTDs worldwide with an estimated 1.5 billion infected people^2^. WHO´s strategy of multi-component integrated management for the control and prevention of STH includes community management through preventive chemotherapy (PC) by mass drug administration (MDA) for communities where prevalence of STH is ≥ 20% as well as preventive measures through the provision of adequate water, sanitation and health education. Currently, the periodic MDA is mostly addressed with the use of donated benzimidazole (BZD) anthelmintic drugs, albendazole (ABZ) and mebendazole^3^.

**Figure 1:**
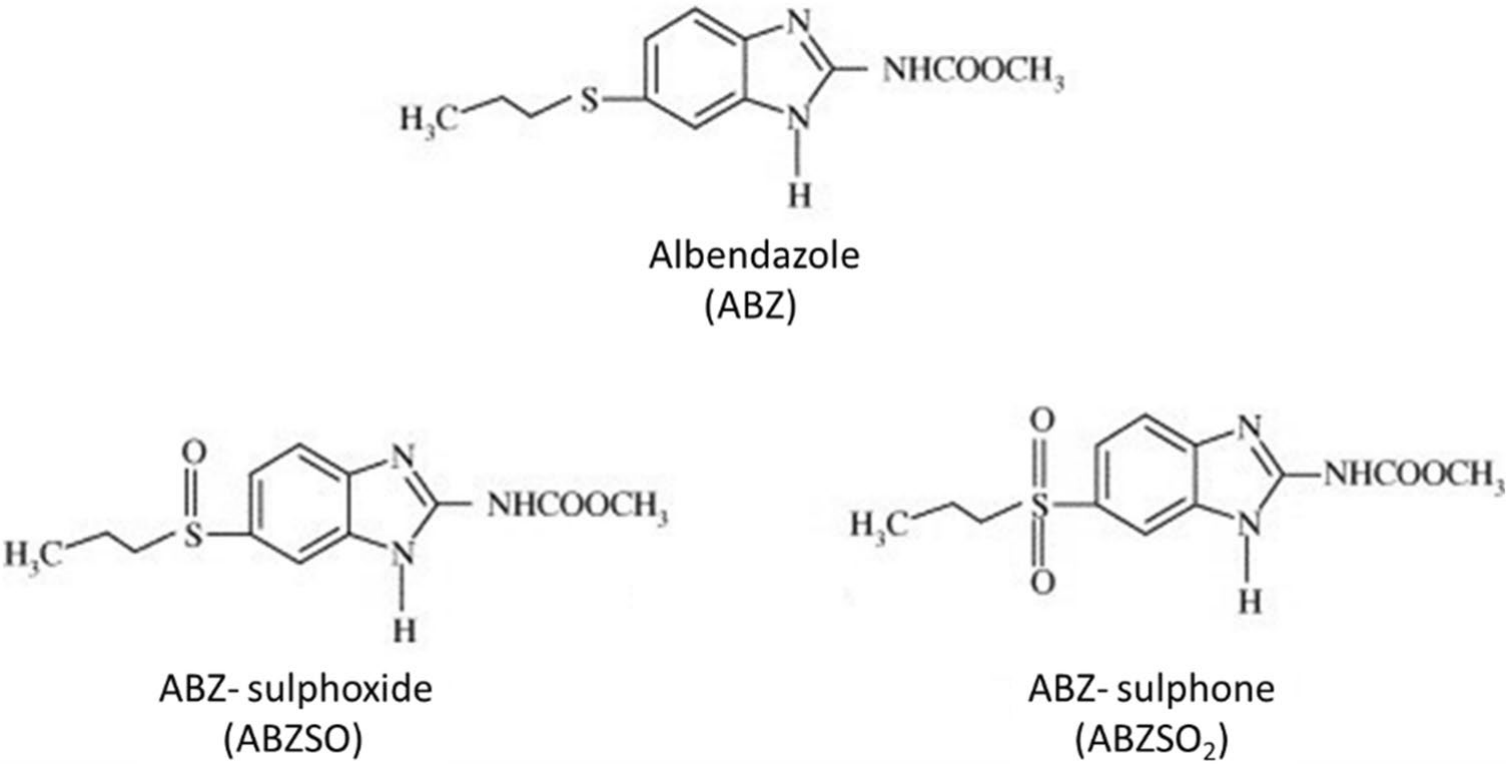
Chemical structure of albendazole (ABZ) and its main metabolites albendazole sulphoxide (ABZSO) and albendazole sulphone (ABZSO_2_).

The oral administration of ABZ implies its dissolution and subsequent absorption in the gastrointestinal tract (GI). Due to extensive pre-systemic metabolism occurring in different tissues^4–6^, ABZ remains undetectable in the systemic circulation of humans^7,8^ and other species^9–11^. Consequently, its metabolites, ABZ sulphoxide (ABZSO) and ABZ sulphone (ABZSO_2_) are the main analytes found in the systemic circulation after ABZ administration. ABZ and its main metabolites structures are shown in Fig. 1. These analytes were also the main ABZ metabolites quantified in human urine samples obtained after ABZ administration^7,8^.

Even with an intensified BZD treatment strategy, certain areas report inadequate treatment efficacy^12,13^ following multiple rounds of MDA. There are several potential reasons for these program failures, which can be attributed to various factors such as: transmission intensities in the local environment, reduced drug efficacy due to suboptimal or poor absorption of the drug formulation, development of resistance to the drugs being used, insufficient program coverage (referring to individuals receiving the drug), lack of compliance with the treatment (meaning individuals not consuming the recommended drug or drug combinations as instructed)^14^.

While the effectiveness and long-term viability of the preventive chemotherapy (PC) strategy are subjects of debate, a notable reduction in the prevalence of moderate and heavy STH infections can predominantly be ascribed to the expansion of drug distribution efforts^15^. To achieve transmission interruption MDA programs require prolonged, high coverage, and treatment compliance, with at least 75–90% of the targeted population being dewormed^3,16^. Nonetheless, in cases where a substantial segment of a community consistently fails to adhere to the treatment, an untreated portion of the parasite reservoir persists, potentially leading to recrudescence through ongoing transmission. This situation diminishes the program's likelihood of achieving elimination^14^. The estimation of coverage/compliance is typically conducted through random cluster surveys^17^, relying on self-reported data from individuals regarding treatment, which are susceptible to both reporter and interviewer bias. Consequently, such methods may not accurately capture the true extent of treatment coverage/compliance^14^.

Improvements of these estimations through more objective methodologies could prove critical in facilitating programs' ability to report accurate coverage/compliance levels. In humans’ studies, the uses of non-invasive samples are very desirable. Facing on that, the detection of ABZ metabolites in urine samples by a HPLC-based methodology was evaluated and validated as an indicator of comply with ABZ treatment to be used in MDA campaigns^8^. The urine-based test would be a significant progress as a non-invasive biological sample to be use programmatically.

Sampling saliva is becoming more popular in the diagnosis and follow-up of diseases, and detection of drugs^18,19^, and its use as a biological sample could be another alternative to estimate compliance after ABZ treatment. Advantages of this biological matrix include its non-invasive nature, its collection without the need for specialized training or equipment, ease of repeat sampling over time, and greater acceptability compared to serum and urine, which can improve the disposition of participants from programs under evaluation.

In that context, this work aimed to characterize the pattern of ABZ and its metabolites excretion in saliva from non-infected human volunteers, to develop a saliva-based biomarker useful to accurately estimate the coverage/compliance in MDA campaigns. In addition, we assessed the comparative blood (dried blood spots -DBS- samples) and saliva disposition kinetics of ABZ and its metabolites. This blood-saliva comparative kinetic description was useful to assess the reliability of saliva concentration profiles as predictors of systemic availability (exposure) for ABZ and its metabolites, and to evaluate if saliva concentrations can be used to discern potential variations in ABZ systemic absorption among individuals.

## Results

The effectiveness of soil-transmitted helminth control programs depends on the treatment compliance by the participant (who actually swallows the drug offered in each round). Estimation of compliance is important in assessing the potential impact of MDA-based control programs. This data is typically conducted through random cluster surveys which are susceptible to both reporter and interviewer bias^14,17^. We focus here on the development and validation of an objective HPLC based-methodology to report accurate compliance levels, throw the detection of ABZ metabolites in saliva samples. The results are mentioned follow:

## Methodology

### Validation results

The HPLC-based methodology employed here was precise, reliable, and simple to quantify ABZ and its metabolites from samples of either saliva or DBS. Tables 1, 2, 3, 4 and 5 shown values of the parameters required for validation according to the ICH Q2(R1). The retention times were: ABZSO, 4.4 min; ABZSO_2_, 6.9 min; ABZ, 11.8 min.

**Table 1:** Absolute recovery (%) of the analytical HPLC method used to quantify albendazole sulphoxide (ABZSO), albendazole sulphone (ABZSO_2_), and albendazole (ABZ) in human saliva and blood (DBS) samples.

Matrix	Analytes	C_nominal_ (µg/mL)	Recovery (%) ± SD^a^		Mean ± SD
SALIVA	ABZSO	0.05	86.1 ± 10.0	90.3 ± 2.7	
		0.5	95.0 ± 7.2		
		1	89.6 ± 5.2		
	ABZSO_2_	0.05	89.8 ± 7.6	88.5 ± 1.4	
		0.5	86.5 ± 5.9		
		1	89.3 ± 8.7		
	ABZ	0.05	70.0 ± 6.2	71.9 ± 2.1	
		0.5	74.2 ± 10.9		
		1	71.6 ± 8.2		
DBS	ABZSO	0.2	94.7 ± 3.0	83.1 ± 11.4	
		0.6	71.9 ± 7.8		
		1	84.7 ± 2.4		
	ABZSO2	0.2	89.8 ± 7.6	88.5 ± 1.4	
		0.6	86.5 ± 5.9		
		1	89.3 ± 8.7		
	ABZ	0.2	91.3 ± 11.8	89.4 ± 2.1	
		0.6	89.7 ± 16.0		
		1	87.1 ± 2.0		

^a^Values are expressed as arithmetic mean (*n* = 6) of samples obtained from different patients. C_nominal_: nominal concentration.

**Table 2:** Interday accuracy of the analytical HPLC method used to quantify albendazole sulphoxide (ABZSO), albendazole sulphone (ABZSO_2_), and albendazole (ABZ) in saliva and blood (DBS) human samples.

Matrix	Analyte	C_nominal_ (µg/mL)	Quantified concentration (µg/mL)^a^	% RE
SALIVA	ABZSO	0.05	0.046	8.00
		0.5	0.541	10.6
		1	0.931	10.9
	ABZSO_2_	0.05	0.044	13.0
		0.5	0.537	9.91
		1	1.077	9.50
	ABZ	0.05	0.054	8.60
		0.5	0.461	10.2
		1	0.957	10.9
DBS	ABZSO	0.2	0.166	16.8
		0.6	0.642	10.5
		2	1.987	3.22
	ABZSO_2_	0.2	0.192	4.17
		0.6	0.558	8.37
		2	2.017	3.83
	ABZ	0.4	0.395	8.02
		0.6	0.664	10.6
		2	2.033	9.17

^a^Values are expressed as arithmetic mean (n = 9). C_nominal_: nominal concentration.

**Table 3:** Interday precision of the analytical HPLC method used to quantify albendazole sulphoxide (ABZSO), albendazole sulphone (ABZSO_2_), and albendazole (ABZ) in saliva and blood (DBS) human samples.

Matrix	Analyte	C_nominal_ (µg/mL)	Quantified concentration (µg/mL)^a^ ± SD	% CV
SALIVA	ABZSO	0.05	0.046 ± 0.002	4.76
		0.5	0.541 ± 0.06	10.1
		1	0.931 ± 0.12	13.6
	ABZSO_2_	0.05	0.044 ± 0.002	4.76
		0.5	0.537 ± 0.054	10.1
		1	1.077 ± 0.110	10.1
	ABZ	0.05	0.054 ± 0.003	5.54
		0.5	0.461 ± 0.06	13.5
		1	0.957 ± 0.161	16.8
DBS	ABZSO	0.2	0.166 ± 0.014	9.51
		0.6	0.642 ± 0.091	11.2
		2	1.987 ± 0.091	4.55
	ABZSO2	0.2	0.192 ± 0.010	5.49
		0.6	0.558 ± 0.097	16.6
		2	2.017 ± 0.116	5.73
	ABZ	0.4	0.395 ± 0.040	10.2
		0.6	0.664 ± 0.035	11.9
		2	2.03 ± 0.242	11.9

^a^ Values are expressed as arithmetic mean (n = 9). C_nominal_: nominal concentration.

**Table 4:** Lower limit of quantification (LLOQ) of albendazole sulphoxide (ABZSO), albendazole sulphone (ABZSO_2_), and albendazole (ABZ), determined in different human biological matrixes: saliva, blood (DBS), and serum.

Matrix		ABZSO	ABZSO_2_	ABZ
Saliva	LLOQ (µg/mL)	**0.01**	**0.05**	**0.05**
	Accuracy (%RE)	10.0	13.0	8.60
	Precision (% CV)	5.70	4.70	5.50
DBS	LLOQ (µg/mL)	**0.1**	**0.1**	**0.4**
	Accuracy (%RE)	13	10.6	8.20
	Precision (% CV)	12.6	12.1	10.1
Serum^1^	LLOQ (µg/mL)	**0.025**	**0.025**	**0.025**
	Accuracy (%RE)	10.3	9.30	18.6
	Precision (% CV)	3.78	8.46	7.42

^1^Data reported in Ceballos et al^8^.

Significant values are in [bold].

**Table 5:** Albendazole sulphoxide (ABZSO), albendazole sulphone (ABZSO_2_), and albendazole (ABZ) stability determined in human saliva under different conditions.

Matrix	Condition	Concentration(µg/mL)	ABZSO	ABZSO_2_	ABZ
			%CV		
Saliva	Freeze/thaw cycles (3x)	0.05	8.93	6.86	7.33
		0.5	12.2	11.5	12.1
		2	5.17	5.06	10.2
	4 °C, 45 days	0.05	10.7	17.9	41.0
		0.5	10.3	15.1	31.4
		2	4.88	10.2	14.5
	− 20 °C,45 days	0.05	15.3	16.2	23.2
		0.5	11.4	19.9	31.5
		2	4.94	11.6	11.4
DBS	rt, 45 days	0.2	9.09	17.5	12.5
		0.6	16.5	9.27	11.2
		2	16.9	10.9	18.8

rt: room temperature.

**Figure 2:**
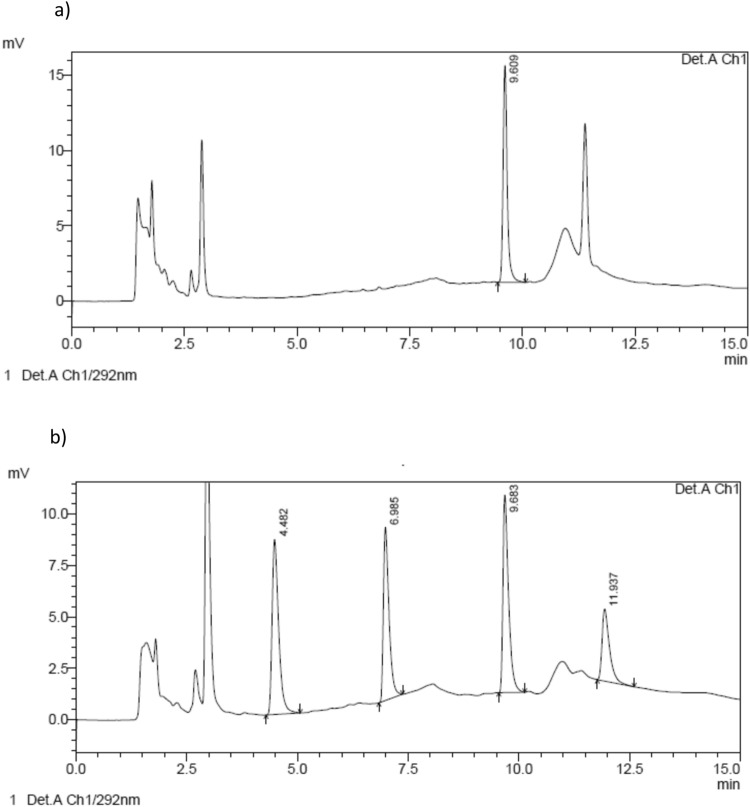
(**a**) Chromatograms obtained from a drug-free saliva sample spiked with oxibendazole used as internal standard (IS); and (**b**) blank saliva sample spiked with albendazole sulphoxide (ABZSO, retention time: 4.4 min), albendazole sulphone (ABZSO_2_, retention time: 6.9 min), IS (9.6 min) and albendazole (ABZ, retention time: 11.9 min).

The blank samples were free of interferences in the period of analytical interest, except in the case of the ABZ sample in DBS in which a slight interference was detected (Selectivity) Blank saliva samples, supplemented with the drug, are shown in Fig. 2.

The Linearity expressed by the correlation coefficient (r2) of the standard calibration curves for ABZ, ABZSO and ABZSO_2_ in saliva; DBS and serum from humans were ≥ 0.995. A very good recovery was obtained for the main metabolites either in saliva or DBS samples, above 88%. The parent drug ABZ showed a better recovery in DBS samples than saliva. The mean absolute recoveries in saliva and DBS matrixes at three concentrations levels are shown in Table 1.

The methods exhibited an acceptable inter-day accuracy and precision in saliva and DBS samples for the three analytes assessed. This was demonstrated by % RE < 16.8% (accuracy) and % CV < 16.8 (precision). The Accuracy and Precision results are shown in Tables 2 and 3, respectively.

The Limit of detection (LOD) in saliva was 0.01 µg/mL for ABZSO, 0.02 µg/mL for ABZSO_2_, and 0.031 µg/mL for ABZ, lower to that determined in DBS samples which was 0.079 µg/mL for ABZSO and ABZSO_2_, and 0.22 µg/mL for ABZ. Similarly, the lower limit of quantification (LLOQ) obtained in saliva was lower to that obtained in DBS samples for the three analytes determined (Table 4). A marked difference in the LLOQ value was observed between DBS and serum samples for the three analytes.

The stability of saliva and DBS samples are shown in Table 5. ABZSO, ABZSO_2_, and ABZ were proven stable in saliva after three freeze/thaw cycles. ABZSO and ABZSO_2_ were stables in saliva samples for 45 days either at 4°C (fridge) or -20°C (frozen) with coefficients of variation (%CV) < 20% for the three concentrations evaluated, which indicates no significant degradation of them in these conditions. Conversely, ABZ at 0.05 µg/mL and 0.5 µg/mL showed % CV > 20% when the long-term stability was determined either at 4 °C or − 20 °C. The DBS samples were stable for 45 days at room temperature, with %CV < 20% for the three analytes.

### Characterization of the in vivo pattern of ABZ/metabolites excretion in saliva. Comparative drug disposition kinetics in saliva and blood (DBS) samples

**Figure 3:**
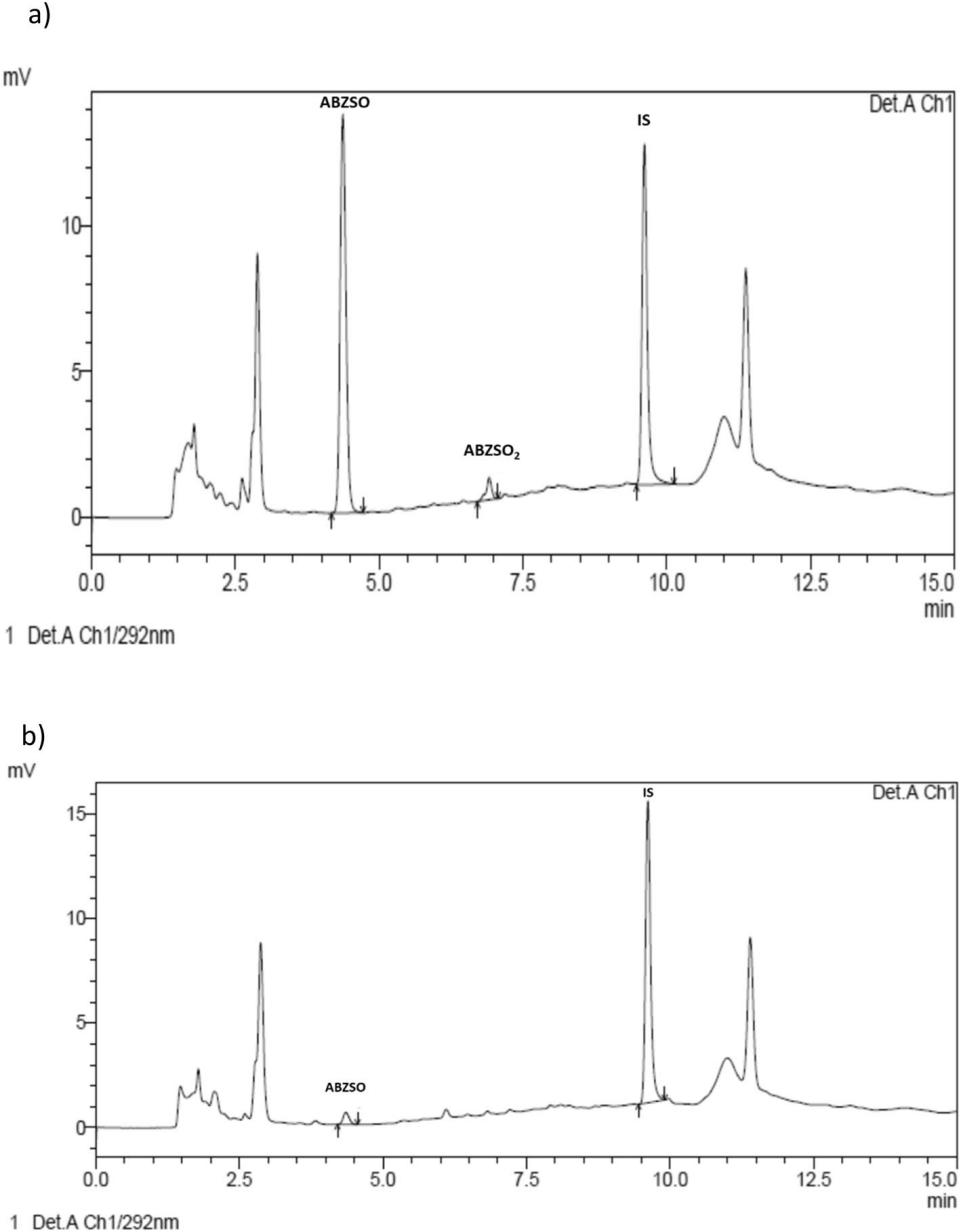
Chromatograms obtained from experimental samples of saliva at 4 and 72 h post ABZ administration to volunteers (**a** and **b**, respectively), ABZSO: albendazole sulphoxide, ABZSO_2_: albendazole solphone, IS: internal standard.

**Figure 4:**
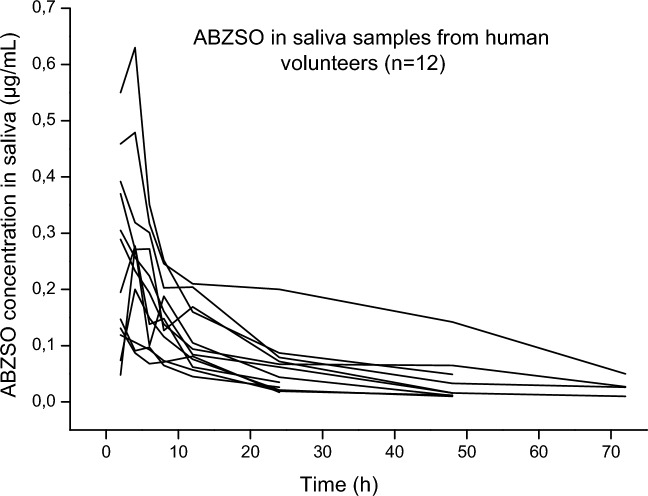
Individual albendazole sulphoxide (ABZSO) concentrations (µg/mL) measured in saliva after the administration of albendazole (ABZ) as a single oral dose (400 mg) to human adult volunteers.

The validated methodologies could be successfully applied to quantify ABZ/metabolites in both saliva and DBS samples from humans orally treated with ABZ (400 mg). Accordingly, to the weight of the twelve volunteers (48–68.4 kg), the dose by body-weight range was between 5.75 to 8.33 mg/kg. Demographic data of individual volunteers are shown in Table 6. After analysis of the experimental samples, ABZ parent drug was not detected in saliva at any time (2–72 h), and only traces of the sulphone metabolite (ABZSO_2_) could be detected below the LLOQ. Conversely, ABZSO was detected over the LLOQ from the first sampling time (2 h) and up to 24 h p.t. in all volunteers, and up to 48 h in the most of volunteers (9/12), while over the whole trial (up to 72 h p.t.) in four of them. Chromatograms obtained from experimental samples 4 and 72 h p.t. are shown in Figs. 2 and 3. The individual concentrations of ABZSO are shown in Fig. 4. The pharmacokinetic analysis in saliva samples was done based on ABZSO concentrations. After ABZ treatment, ABZSO reached a Cmax (0.30–0.15 µg/mL) in saliva at 2.8–1.03 h, denoting the rapid absorption and metabolism of ABZ. The AUC_LOQ_ was 4.73–3.31 µg.h/mL, and the chosen sampling schedule provided a reliable estimation of the extent of exposure since AUC_0-LOQ_ covers ≥ 80% of AUC_0-∞_ (AUC extrapolated to infinity). The mean (- SD) pharmacokinetic parameters for ABZSO in saliva are summarized in Table 7. High inter-individual variability was observed in the main pharmacokinetic values. However, when these values were normalized by dose (mg/kg) it was observed a decrease in the CV (%) for Cmax (50 to 41%) and AUC_0-LOQ_ (69 to 57%) compared to raw data.

**Table 6:** Demographic Data of Study volunteers.

Volunteers	FG01	NE02	AL03	MJ 04	YN05	AJ06	RF07	VC08	SE09	IC10	DR11	AR12
Age (years)	33	39	20	21	27	25	23	28	34	25	33	28
Gender	F	F	M	M	F	F	M	M	F	F	M	M
Weight (kg)	48	50.1	68.4	69.5	51.1	64	67.9	53	62.3	56	68.5	68.4
Height (cm)	159	150	183	174	162	167	175	164	159	160	168	174
Body Mass Index (kg/m^2^)	18.9	22.3	20.4	22.9	19.5	22.9	22.2	19.7	24.6	21.8	24.3	22.6

F: female; M: male.

**Table 7:** Mean pharmacokinetic parameters (± SD) of albendazole sulphoxide (ABZSO) quantified from saliva and DBS after administration of albendazole (ABZ) to healthy human volunteers.

PK parameters	Saliva		Blood (DBS)	
	Mean (range)	SD	Mean (range)	SD
Cmax (µg/mL)	0.30 (0.11–0.63)	0.152	0.62* (0.39–1.00)	0.191
Tmax (h)	2.83 (2–4)	1.030	4.00	0.000
AUC_LOQ_ (obs) µg.h/mL	4.73 (1.7–12.5)	3.318	8.43* (3.50- 19.0)	4.529
AUC∞ (area) µg.h/mL	5.36 (1.7–14.5)	4.652	10.5* (4.00–28.0)	6.433
MRT (area)	18.0 (10.8–30)	6.432	14.8 (6.80–28.0)	5.620
Half-life el (h)	13.9 (7.2–28.7)	5.547	14.5 (3.60–37.0)	9.655
Half-life form (h)	1.88 (0.745–4)	1.149	1.20 (0.36–2.6)	0.628
Norm Cmax (µg/mL)	0.26 (0.10–0.46)	0.107	0.54* (0.30–0.73)	0.141
Norm AUC_0-LOQ_ (µg.h/mL)	3.95 (1.20–8.70)	2.264	7.60* (3.50–18.40)	4.335

Cmax: maximum concentration; Tmax: time to reach Cmax; AUC_0–LOQ_ area under the curve concentration vs time from time zero to the limit of quantification; AUC∞ area under the curve concentration vs time from time zero extrapolated to infinity; MRT: mean residence time; Half-life el: elimination half life; half-life form: formation half life. *Statistical differences (*P* < 0.05) between biological matrixes (Saliva and DBS).

The pharmacokinetic analysis of ABZ and its metabolites in DBS samples was also based on ABZSO metabolite concentration, since only traces of the ABZSO_2_ could be detected in some volunteers, all of them below of LLOQ. ABZSO could be accurately identified from the DBS samples mostly up to 24 h (11/12 volunteers). In 5 volunteers, this methodology allowed the detection of the ABZSO for up to 48 h. In all cases, the ABZSO concentrations at 72 h were below the LLOQ (0.1 µg/mL). The mean (- SD) pharmacokinetic parameters for ABZSO in DBS samples are summarized in Table 7. In all volunteers, the peak DBS concentration (0.624–0.02 µg/mL) was observed at 4 h after ABZ treatment. This value decreased to 0.54–0.14 µg/mL when the dose was normalized by weight. The AUC_LOQ_ was 8.80–4.54 and cover 80% of AUC_0-∞_. Similar ABZSO concentrations (*P* < 0.05) were observed at 2, 4, and 8 h post-ABZ treatment after extraction from DBS or serum samples. A positive correlation coefficient was obtained between these matrixes (Pearson r-value: 0.907, *P* < 0.05). However, the analyte was longer detected above the LOQ (0.025) in the serum samples (up to 72 h in 9 of 12 volunteers) (Table 8).

**Table 8:** Mean (± SD) concentrations obtained of albendazole sulphoxide (ABZSO) extracted from DBS or serum samples of volunteers treated with albendazole.

Biological matrix	Time post-treatment (h)			
	2	4	8	72
DBS (µg/mL)	0.448 ± 0.28	0.580 ± 0.21	0.338 ± 0.15	< LOQ
Serum (µg/mL)	0.434 ± 0.28	0.604 ± 0.28	0.373 ± 0.14	0.067 ± 0.05

No statistical differences (P > 0.05) was obtained between ABZSO concentrations in blood (DBS) and serum at 2, 4, and 8 h post-treatment.

**Figure 5:**
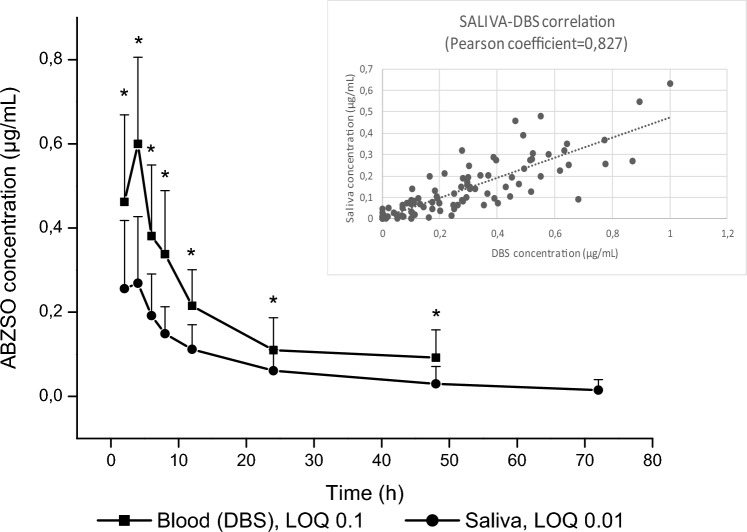
Comparative albendazole sulphoxide (ABZSO) blood (DBS) and serum concentration profiles (mean ± SD) obtained from albendazole (ABZ) treated human volunteers (*n* = 12). * Statistically significant differences (*P* < 0.05). The insert shows a positive correlation was observed between the ABZSO concentration in saliva and DBS samples.

Figure 5 shows the comparison between ABZSO concentrations vs time profiles obtained in saliva and DBS samples. The concentration profiles detected in the DBS samples were higher (*P* < 0.05) than that in saliva from 2 to 48 h. As we show in Table 7, Cmax was reached in saliva faster than in blood (in which the Tmax was 4 h for all of volunteers). These differences could be explained by the type of sampling, the range of sampling times that may give some bias in the results. Increasing the frequency of sampling could provide more accurate and detailed insights into the specific time at which the maximum concentration (Cmax) is achieved. However, with the methodologies validated here, this analyte could be measured longer in saliva (above its LOQ). A positive correlation was observed between the ABZSO concentration in saliva and DBS samples with a Pearson coefficient value of *r* = 0.827 (*P* < 0.05) considering all sampling points (2–72 h).

## Discussion

Interrupting or reducing STH infections, which affect 1.5 billion individuals worldwide^2,20^, implies dealing with several issues in addition to WASH (water supply, sanitation, and hygiene). These include improvement of drug efficacy, determining who should be treated, and ensuring coverage. The coverage target across the different age classes have a considerable impact on the STH infections levels. Achieving high coverage can indeed pose various social, logistical, and technical challenges^21^. However, even when coverage levels reach a significant fraction of the target population if a proportion of a community fails to comply with chemotherapeutic treatments, a fraction of the parasite population remains untreated as a reservoir allowing continued transmission. That is thought to be a major obstacle to the elimination of STH by mass drug administration (MDA)^22^. Recent modeling suggests that transmission interruption may be possible through high levels of coverage (community-wide delivery of MDA) and compliance (individuals actually consuming the drug)^14,23^. Consequently, it is critical to improve the tools for estimating compliance or treatment adherence by enhancing data collection and reporting mechanisms, as well as developing new strategies to address this question.

Blood is the conventional biological matrix to measure the concentration of drugs in the body. Both plasma and serum can be analyzed to determine the amount of a drug present in the systemic circulation, including ABZ and its metabolites. However, the invasive nature and clinical conditions necessary for sampling have led to ABZ human pharmacokinetic (PK) studies being performed primarily in healthy adults, with limited cases involving infected adults or children^24^. Consequently, the feasibility of alternative biological matrices, for examining drug pharmacokinetics have explored^25^. In agreement with that, we have previously characterized the ABZ and its metabolites excretion in urine and developed reported a urine based-methodology useful to accurately determine the presence or not of ABZ metabolites in human samples, even 48 h after its assumed ingestion^26^. Urine, being a non-invasively collected sample, is valued for its ability to detect individuals who received or not their treatments. In addition, saliva sampling may offer another non-invasive and convenient method for monitoring drug levels and treatment coverage. The potential advantages of a saliva-based methodology (compared to urine) lie on the saliva sample is easy to collect, well tolerated and preferable in studies where reducing participant discomfort is crucial; the volume of saliva required for donation is smaller, and it is more convenient for obtaining samples from children. There are a substantial body of evidence supports the notion that saliva serves as a valuable matrix for therapeutic drug monitoring^27^. This study reports the validation of a HPLC-method developed to quantify the concentration of ABZ and its metabolites in saliva. And then this method was applied to investigate the disposition kinetics of ABZ metabolites in saliva obtained from healthy volunteers.

The selectivity is the capacity of an analytical method to differentiate between the analyte under consideration and any other substances that might exist in the sample under analysis. Similarly to what was observed in urine^26^, saliva samples exhibited no interference in the retention time of the ABZSO metabolite chromatographic peak. Moreover, the methodology demonstrated consistent sensitivity in both urine and saliva matrices, as indicated by the lower limit of quantification (LLOQ), set at a minimum concentration of 0.01 µg/mL in the calibration curves. This allowed for the detection of the ABZSO metabolite (the main metabolite detected in both biological matrices) for 48 h after ABZ administration in the most of the treated volunteers. Notably, a significant difference between the two developed methodologies is the lower sample volume required for saliva (0.25 mL) compared to urine (5 mL), facilitating the storage and processing of samples, which is considered a great practical/logistic advantage.

The developed saliva-based methodology would allow the community health workers and program staff to take unstimulated saliva samples up to 24 h after ABZ administration and accurately determine the presence or absence of the ABZSO metabolite. This assessment can help evaluate compliance after ABZ mass deworming campaigns.

Taking into account all aforementioned considerations, and in the context of mass drug administration activities carried out in endemic regions, implementing these methodologies can pose challenges in sample collection, storage, and transportation of either saliva or urine samples, which constitute potential hurdles in the process. Economic constraints, high population density, and lack of household tenure would favor the choice of collecting saliva, overcoming potential cultural and privacy barriers associated with urine collection. In addition, the logistical challenges associated with the storage and transportation are mitigated, since the developed methodology is based on a minimal volume (capable of being transported in a 1.5 mL tube) and the chemical stability determined for that sample including its long stability (45 days) at 4 °C, would allow the use of this methodology in areas with poor infrastructure, by using a portable refrigerator system. The collection of saliva is usually less invasive and more comfortable for participants compared to collecting urine samples, which can improve acceptability by end users^28,29^. This strategic shift toward saliva-based sampling not only streamlines operational aspects but also contributes to its overall feasibility and effectiveness in resource-limited settings.

Good correlations between ABZSO concentrations in saliva, capillary blood (DBS), and serum samples has been determined here. As reported for others orally administered drugs^30,31^. Casati et al^32^. have recently conducted a review that specifically compares drug detection in saliva (oral fluid) and blood samples. The study explores the utility of saliva as a tool for assessing compliance, monitoring, and evaluating the presence of drugs of abuse, both during pre-anesthetic assessment and in emergency room scenarios. The authors concluded that a substantial number of studies consistently highlight a robust correlation between the two biological fluids. Consequently, saliva emerges as a viable alternative to blood, particularly for long-term surveillance (e.g., therapeutic drugs) or for screening a large number of patients.

Overall, the work reported here could contribute to improving the outcome of ABZ-based parasite control programs, since reports for the first time the in vivo pattern of ABZ metabolites excretion in saliva. The full development of the analytical methodology to measure ABZ metabolites in saliva, the assessment of drug chemical stability in saliva, and the in vivo measurement of drug/metabolites in saliva samples of ABZ-treated volunteers, offer strong scientific evidence to propose the use of saliva as a valid biological sample to assess compliance in MDA programs. Nevertheless, the study is constrained by the limited number of individuals and the standardized assay conditions, encompassing factors such as age, formulation quality, health conditions, and treatment, among others. These factors may vary in real-world field conditions, potentially impacting the drug's pharmacokinetics and its subsequent profiles in saliva. As a next step, it is essential to evaluate the methodology under more realistic field conditions, incorporating a larger and diverse populations and program settings. The acceptability of it has to be evaluated at all levels of the healthcare system as well as with the individuals at the communities where objective measurements of coverage are needed. Adding the cost of sample collection, storage, transportation and processing is a critical challenge; however, the implications of accurate measurements of coverage can contribute to optimize the efforts by identifying programmatic difficulties and adjusting the resources to problematic areas; which might ultimately result in cost savings. Systematic non-compliance is another aspect that requires field validations and is a potential limitation of this approach^22^, since there is a risk that the same factors affecting the compliance to medication, compromise participation in sample collection.

## Materials and methods

All methods were carried out in accordance with relevant guidelines and regulations.

### Ethics and study population

The study protocol and Informed Consent Form (ICF) were approved by the Bioethics Committee at the Comisión Provincial de Investigaciones Biomédicas-Ministerio de Salud Pública de la Provincia de Salta (Argentina). This trial was conducted at the facilities of the Instituto de Investigaciones de Enfermedades Tropicales, Universidad Nacional de Salta, Orán, Argentina. Volunteers provided written consent for their participation in the study before any study procedures. The trial is registered at clinicaltrial.gov (12/07/2022, NCT05453045).

Adults age between 18 and 39 (6 female and 6 male), with an average weight of 60.6 kg (range 48–69.5 kg) from the region of San Ramon de la Nueva Orán, were enrolled in the study. All participants completed the study visits and tolerated the dose of ABZ uneventfully. The sample size calculation for this trial was based on the area under the concentrations versus time curve (AUC) data from a previous trial on serum and urine ABZ kinetics in human volunteers^8^.

Inclusion criteria targeted males and females 20 to 39 years old (both inclusive), with body mass index 18–24.9, without a significant medical history; women of childbearing age with a negative pregnancy test before entering the study and practicing effective contraceptive precautions during their participation in the study and for up to 30 days after its completion. Exclusion criteria included intake of ABZ or other BZD drugs within the last 30 days, malabsorption or other syndromes that could compromise the tolerability or absorption of ABZ, history of hypersensitivity or intolerance to ABZ or its inactive ingredients, presence of acute or chronic conditions, pregnancy or breast feeding and those who have participated in clinical pharmacokinetic studies in the last 3 months. Liver function tests and hemoglobin were assessed at baseline.

### Characterization of the in vivo pattern of ABZ/metabolites excretion in saliva

#### Comparative drug disposition kinetics in saliva and blood (DBS) samples

Each volunteer was treated with a single oral dose of ABZ (400 mg, Nematel, Elea Argentina) 30 min after receiving a standard meal (estimated fat content: 15 g). Serial saliva (1 mL) and dried blood spots (DBS) samples were taken pre-dose and at 2, 4, 6, 8, 12, 24, 48, and 72 h post-ABZ treatment (p.t.). In addition, venous blood samples were collected at 2, 4, 8, and 72 p.t. and centrifuged immediately to obtain serum samples which were stored at − 70 ºC until assayed. These samples were collected to assess possible discrepancies between ABZ/metabolites concentration in DBS and serum. Saliva samples were collected in plastic vials and stored at − 70 ºC until assayed. The micro sampling (DBS) was performed by taking capillary blood from each individual following the procedure previously described^33^. Two blood drops at each time point were collected from each participant in DBS cards (Western blotting filter paper, Thermo Scientific, USA). The DBS cards were allowed to dry for at least 2 h, and then stored at room temperature in sealed plastic bags until analysis by HPLC.

#### Chemicals

ABZ, ABZSO, ABZSO_2_ and oxibendazole (OBZ, internal standard) from Sigma–Aldrich, St. Louis, MO USA (99% purity). Stock and working solutions of a mix of the pure reference standards (ABZ + ABZSO + ABZSO_2_) were prepared in methanol. Acetonitrile and methanol were HPLC grade from J.T. Baker (Mallinckrodt Baker, Mexico). Water was distilled and deionized using a water purification system (Simplicity®, Millipore, São Paulo, Brazil).

## Analytical phase

### Validation of the analytical methodology in saliva and DBS samples

Full validation of the analytical procedures for the extraction and quantification of each molecule (ABZ, ABZSO, ABZSO_2_ and OBZ) in each biological matrix (saliva and DBS samples) was performed before the analysis of the experimental samples, following internationally recognized criteria^34^. The analytical procedures for the extraction and quantification of ABZ, ABZSO, and ABZSO_2_ in serum samples were validated and described previously by our group^8^.

The calibration curves and quality control (QC) samples were prepared with drug-free human saliva, serum, and blood donated by participant volunteers. For DBS validation, 70 µL of the obtained blood (drug-free and supplemented with ABZ/metabolites working solutions to achieve the final concentration used in calibration curves) were dropped onto the DBS cards. The dry DBS cards were stored at room temperature in sealed plastic bags until the drug extraction and analysis by HPLC. To validate the methodology for the DBS and saliva kinetic studies, the following parameters were determined: Selectivity, Limit of Detection (LOD), Lower Limit of Quantification (LLOQ), Linearity, Recovery, Accuracy, Precision, and Stability.

*Selectivity and sensibility*: Blank saliva and DBS human samples obtained from six different human sources were fortified with Internal Standard (IS) to determine the limit of detection (LOD) and the lower limit of quantification (LLOQ) from each matrix. The Limit of Detection (LOD) was estimated by integrating the baseline noise of the HPLC system in the area covering the mean retention time (RT) of each analyte and was defined as the mean baseline noise/IS peak area ratio plus three standard deviations (SD). The *Lower Limit of quantification* (LLOQ) was defined as the lowest drug concentration (*n* = 6) on the DBS/saliva standard curve that could be quantified with a precision not exceeding 20% and accuracy within 20% of the nominal concentration.

*Linearity*: Although the methodology to quantify ABZ and its metabolites in serum was previously validated, the linearity parameters were determined once again. The linearity was tested by constructing calibration curves for each compound in saliva, DBS and serum. The calibration ranges for ABZ, ABZSO, and ABZSO_2_ were the following: for saliva the range was 0.01–2 µg/mL, using 6 different concentrations (0.01, 0.05, 0.1, 0.5, 1, and 2 µg/mL, *n* = 3); for DBS the range was 0.1–2 µg/mL, using 6 different concentrations (0.1, 0.2, 0.4, 0.6, 1, and 2 µg/mL, *n* = 6); and for serum the range was 0.02–2 µg/mL, using 6 different concentrations (0.02, 0.05, 0.1, 0.5, 1, and 2 µg/mL, *n* = 3).

The data were analysed for linearity using the least-squares regression method, using the Run Test and ANOVA to determine if the data differed from a straight line.

*Recovery:* The extraction efficiency of the analytes under study was determined by comparison of the detector responses (peak areas) obtained from QC samples (low, medium and high, *n* = 6) with the peak areas resulting from direct injections of equivalent concentrations of analytes prepared in methanol.

*Accuracy and precision:* These parameters were determined by evaluation of three QC concentrations (low, medium, and high) each prepared in three replicates of DBS/saliva samples and running across three consecutive working days (three validation sets each day). Accuracy of the method was measured by the differences between nominal and calculated concentration (Cq) obtained in different days and expressed as the relative error (% RE), this was considered acceptable when the %RE was ≤ 20. Precision was expressed as the coefficient of variation (% CV), and was considered acceptable when the %CV was ≤ 20%.

*Stability: *The stability of saliva samples was determined at different field and lab conditions. Three QC samples (low, middle and high concentration, six replicates) not extracted were maintained at − 20 °C and freeze/thaw for three consecutive days (freeze/thaw cycles) or at 4 and − 20 °C for 45 days (long-term stability) before extraction of analytes. Stability was determined as the coefficient of variation (%CV) between analysed samples. In addition, DBS samples long-term stability for 45 days was determined by testing three QC samples (low, middle and high concentration, six replicates each, not extracted) to evaluate their stability when no freezer is available in the field. Stability was considered acceptable if the mean concentration obtained at the specified time point agrees with those of the freshly prepared QC samples (six replicates) at the same concentrations within—20%. Stability was determined as the coefficient of variation (% CV) between analysed samples.

## Saliva, dried blood spot (DBS) and serum sample process

### Saliva sample extraction

Experimental and fortified (for validation) samples of saliva (250 µL) were spiked with 10 µL of OBZ as IS (5 µg/mL) and then added with 1 mL of water. The total sample was transferred into a Supelclean LC_18_ cartridge (RP-18, 100 mg, Strata®, Phenomenex, CA, USA) previously conditioned. After washing with deionized water (1 mL) followed by 1 mL water–methanol (4:1 v/v), the cartridges were dried off for 5 min. Finally, samples were eluted with acetonitrile (2 mL) and the eluted was evaporated to dryness under a gentle stream of nitrogen at 56 °C in a water bath (Zymark TurboVap LV evaporator, American Laboratory Trading, Inc., Lyme, CT, USA).

### Dried blood spot (DBS) sample extraction

DBS experimental (whole dried drop) and fortified samples were punched from the card and transferred to a polypropylene tube (5 mL). The samples were spiked with 10 µL of IS (5 µg/mL). The methodology used for ABZ/metabolites extraction from DBS was previously described by Matamoros et al^35^. Briefly, samples were added with 1 mL of acetonitrile/water (4:1 v/v), followed by shaking (15 min), sonication (90 min), and centrifugation. The liquid fraction was transferred to a 5-mL glass tube and evaporated to dryness.

### Serum sample extraction

Serum experimental and fortified samples (250 µL) were spiked with 10 µL of IS (OBZ, 40 µg/mL), and diluted with 0.5 mL of water. Drug molecules were extracted by a solid phase extraction using C_18_ cartridges in a manifold vacuum. The sample was applied and then sequentially washed with 2 mL of HPLC water, dried 5 min, and finally eluted with 3 mL of methanol. The eluted was evaporated to dryness under a gentle stream of dry nitrogen at 56 °C in a water bath.

The dry residue of either blood, saliva or serum was dissolved in 150 µL of mobile phase (acetonitrile:water, 27:73) and shaken (15 min) before injection into the chromatographic system.

### HPLC System and chromatographic conditions

Experimental and fortified saliva, DBS, and serum samples were analyzed for ABZ, ABZSO, and ABZSO_2_ by HPLC. After extraction, 50 µL of the sample was injected into a Shimadzu Chromatography System (Shimadzu Corporation, Kyoto, Japan). HPLC equipment composition and settings was described by Ceballos et al^8^, elution from the stationary phase was carried out at a flow rate of 1.2 mL/min using a mobile phase based on acetonitrile and ammonium acetate buffer (0.025 M, pH 6.6) as the mobile phase. The C_18_ reversed-phase column (5 µm, 250 mm × 4.6 mm) was Kromasil (Kromasil®, Sweden). The compounds were identified with retention times of 99% pure reference standards.

### Pharmacokinetics analysis

The pharmacokinetic parameters peak concentration (Cmax), time to peak concentration (Tmax), area under the concentration time-curve (AUC), elimination half-life (T½el) and metabolite formation (T½for) and the mean residence time (MRT) were obtained using the program PK Solution (Summit Research Services, Ashland, USA). The pharmacokinetic analysis was performed using non-compartmental (area) and compartmental (exponential terms) methods without presuming any specific compartmental model.

### Statistical analysis of the data

The pharmacokinetic parameters and concentration data are reported as arithmetic mean—SD. The Pearson correlation coefficient was used to determine the correlation between ABZSO concentration in either saliva and DBS or saliva and serum. The statistical analysis was performed using the Instat 3.0 Software (Graph Pad Software, CA, USA). Normalized Cmax and AUC_0-LOQ_ were used to compare differences among groups, and were calculated for each individual volunteer (IV) as follows:

$${\text{Normalized Cmax}} = \, \left( {{\text{Cmax IV }}*{ 5}.{75}} \right) \, /{\text{ IV dose}}$$

$${\text{Normalized AUC0-LOQ}} = \, \left( {{\text{AUC IV }}*{ 5}.{75}} \right) \, /{\text{ IV dose}}$$

where Cmax/AUC_0-LOQ_ IV is the value of these parameters calculated for each IV, 5.75 is the lowest administered standard dose (mg/kg) among volunteers and IV dose is the standard dose (mg/kg) received for each IV.

## Data Availability

All relevant data are within the paper.
